# Spatial gender-age-period-cohort analysis of pancreatic cancer mortality in Spain (1990–2013)

**DOI:** 10.1371/journal.pone.0169751

**Published:** 2017-02-15

**Authors:** Jaione Etxeberria, Tomás Goicoa, Gonzalo López-Abente, Andrea Riebler, María Dolores Ugarte

**Affiliations:** 1 Department of Statistics and Operations Research, Public University of Navarre, Pamplona, Spain; 2 Institute for Advanced Materials, InaMat, Public University of Navarre, Pamplona, Spain; 3 Consortium for Biomedical Research in Epidemiology and Public Health (CIBERESP), Madrid, Spain; 4 Research Network on Health Services in Chronic Diseases (REDISSEC), Madrid, Spain; 5 Institute of Health Carlos III, Madrid, Spain; 6 Department of Mathematical Sciences, Norwegian University of Science and Technology, Trondheim, Norway; Stony Brook University, Graduate Program in Public Health, UNITED STATES

## Abstract

Recently, the interest in studying pancreatic cancer mortality has increased due to its high lethality. In this work a detailed analysis of pancreatic cancer mortality in Spanish provinces was performed using recent data. A set of multivariate spatial gender-age-period-cohort models was considered to look for potential candidates to analyze pancreatic cancer mortality rates. The selected model combines features of APC (age-period-cohort) models with disease mapping approaches. To ensure model identifiability sum-to-zero constraints were applied. A fully Bayesian approach based on integrated nested Laplace approximations (INLA) was considered for model fitting and inference. Sensitivity analyses were also conducted. In general, estimated average rates by age, cohort, and period are higher in males than in females. The higher differences according to age between males and females correspond to the age groups [65, 70), [70, 75), and [75, 80). Regarding the cohort, the greatest difference between men and women is observed for those born between the forties and the sixties. From there on, the younger the birth cohort is, the smaller the difference becomes. Some cohort differences are also identified by regions and age-groups. The spatial pattern indicates a North-South gradient of pancreatic cancer mortality in Spain, the provinces in the North being the ones with the highest effects on mortality during the studied period. Finally, the space-time evolution shows that the space pattern has changed little over time.

## Introduction

The interest in studying pancreatic cancer mortality has increased in the last years due to its high lethality. The reason is that during the early stages of the disease (when the tumor is more treatable) there are usually no symptoms and therefore this cancer is typically diagnosed at a late stage. Regarding the last estimates provided by GLOBOCAN in 2012 [1] pancreatic cancer was the 12th most common cancer worldwide, with 337,872 new cases (2.4% of the total of cancer) and 330,391 deaths (4% of the total of cancer). The high lethality makes pancreatic cancer incidence rates similar to mortality rates (4.2 and 4.1 cases respectively per 100,000 inhabitants worldwide in 2012) and therefore, mortality rates become a good indicator of incidence. This fact is relevant in Spain as there are not cancer incidence registers in all Spanish regions and then the study of cancer incidence in small areas becomes very difficult.

Pancreatic cancer is more common in males than in females and mortality rates increase with age. The American Cancer Society [2] in its web site indicates that almost all patients are older than 45 years and about two-thirds are at least 65 years old. In Spain, the average age at death for people having pancreatic cancer is 68.5 in women and 70.9 in men (information corresponding to year 2014) [3]. On the other hand, males are about 30% more likely to develop pancreatic cancer than females. Some other studies concluded that the age-standardized mortality rates due to pancreatic cancer showed an increasing trend in the last decades in most of the countries worldwide [4] and this increasing trend was also found in Spain [5]. The analysis of pancreatic cancer mortality rates over time is usually not sufficient to identify possible determinants of the disease. Period effects could be affected by improved methods of diagnosis, treatments, or even the disease codification. In this context, the analysis of the cohort of birth could add extra information related to generation effects reflecting environmental and lifestyle risk factors in the early periods of life [6].

In 2006 the Institute of Health Carlos III of Spain published an Atlas of Cancer Mortality in small areas (municipalities) in Spain using data between 1989 and 1998 [7]. In this publication, a strong geographical pattern of pancreatic cancer mortality was shown. These results indicated a high risk in northern Spain, covering almost all of the municipalities of the provinces of Cantabria and the Basque Country, and a very important part of Navarre, La Rioja, east of Asturias, and northern León and Palencia. There was also a second hot spot in the South-West of Spain. However, knowledge of the evolution of this geographical distribution over time is scant. In addition, as far as we know, there are no studies analysing jointly the effects of age, period, birth cohort, and geographical distribution on pancreatic cancer mortality.

The goal of this paper is to conduct a spatial, gender, age, period, and birth cohort analysis to provide estimates of pancreatic cancer mortality rates in 50 provinces of Spain using the most recently available data that corresponds to the period 1990–2013. Some multivariate gender-age-period-cohort models including spatial effects will be evaluated. The models permit to understand the distribution of mortality rates by gender, the spatial distribution of rates, and their evolution according to age, period, and cohort of birth.

## Materials and methods

Official death certification data from pancreatic cancer and population were obtained from the Spanish National Statistical Institute (INE). Data were organized by region (fifty Spanish provinces), year (from 1990 to 2013), age-groups [25–30), [30–35), …, [80,85), [85+), and gender. Pancreatic cancer mortality cases were coded as 157 and C25 according to the 9th and 10th revision of the International Classification of Diseases, respectively. The number of age-specific deaths and the corresponding population for those age groups for the whole period in Spain are given in Table 1.

**Table 1:** Age-specific pancreatic cancer deaths and the corresponding aggregated population by gender in the period (1990–2013) in Spain.

	Females		Males	
Age-group	Cases	Population	Cases	Population
[25, 30)	29	39,112,750	30	40,674,966
[30, 35)	78	40,479,316	127	42,050,505
[35, 40)	208	39,037,967	343	40,159,381
[40, 45)	456	36,471,240	950	36,921,758
[45, 50)	886	33,664,579	1,819	33,514,408
[50, 55)	1,477	30,392,137	3,165	29,678,151
[55, 60)	2,339	28,256,099	4,751	26,894,691
[60, 65)	3,631	26,988,257	6,563	24,844,905
[65, 70)	5,254	25,364,551	8,319	22,216,742
[70, 75)	7,052	22,899,701	9,015	18,417,269
[75, 80)	8,999	19,621,682	8,729	13,916,416
[80, 85)	8,764	14,420,414	6,328	8,697,951
[85, +)	9,173	12,417,814	4,513	5,711,393
**Total**	48,346	369,126,507	54,652	343,698,536

**Fig 1:**
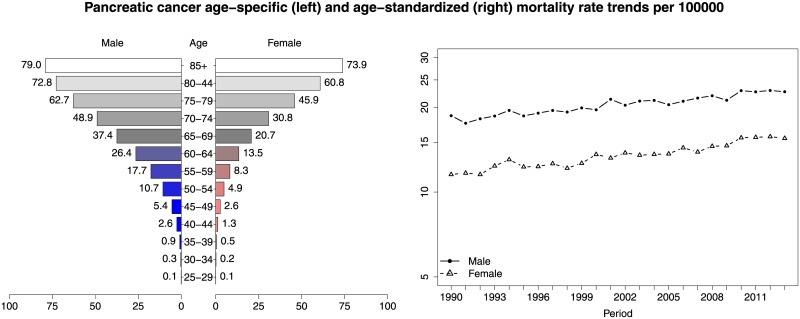
Age-specific mortality rates per 100,000 by gender (left), and age-standardized rate trends between 1990–2013 by gender on semi-logarithmic scale on the y-axis (right).

In a first preliminary analysis, age-specific mortality rates as well as age-standardized mortality rate trends by gender were computed per 100,000 inhabitants (see Fig 1). Age-standardized rates were calculated using the direct method and the revised standard European population 2013 [8]. Later, a set of statistical models [9] were considered to analyze several effects jointly. More precisely, models combining gender, region, age-group, period, and cohort as well as space-cohort and space-time interactions were considered. Model fitting and inference were carried out using Bayesian methodology, specifically, integrated nested Laplace approximations (INLA) (see [10]). The technique is implemented in the software R through the package R-INLA (www.r-inla.org). Finally, to select the best model among the different proposals, the WAIC [11] and the DIC information criteria [12] were computed. In what follows a detailed description of the final selected model is provided. Conditional on the mortality rate *r*_*gait*_, the number of deaths *C*_*gait*_ is assumed to be conditionally independent Poisson distributed, i.e.

$$C_{gait}|r_{gait}∼Poisson\left(\mu_{gait}=n_{gait}r_{gait}\right),\;\log\mu_{gait}=\log n_{gait}+\log r_{gait},$$

where the term log *n*_*gait*_ is an offset (population counts in each gender *g*, area *a*, age-group *i* and year *t*) and log *r*_*gait*_ is modeled as

$$\log r_{gait}=\beta_{g}+\alpha_{tg}+\gamma_{ig}+\kappa_{kg}+\phi_{a}+\delta_{at},$$ (1)

where *β*_*g*_ is a gender-specific intercept, *α*_*tg*_, *γ*_*ig*_ and *κ*_*kg*_ represent the gender specific temporal, age, and cohort effects respectively. The cohort index *k* is given by *k* = *M* × (*I* − *i*) + *t*, where I = 13 is the number of age-groups, and M = 5 as age group intervals are five times wider than period intervals [9, 13]; *ϕ*_*a*_ is a spatially structured effect with the so called intrinsic autoregressive prior distribution (ICAR) given by Besag [14], and *δ*_*at*_ is a random effect representing a spatio-temporal interaction that belongs to the class of interactions described by Knorr-Held [15]. For a detailed definition of Model (1), prior distributions, sensitivity analysis, and identifiability issues of APC models see S1 Appendix.

## 1 Results

From 1990 to 2013, a total of 102,998 pancreatic cancer related deaths were registered in Spain, 48,346 (46.94%) were females and 54,652 (53.06%) were males. Age-specific rates by gender are shown in Fig 1 (left). Clear differences between males and females are observed. As expected, mortality rates increase with age and are greater in males than in females. In Fig 1 (right) age-standardized mortality rate trends between 1990–2013 by gender are also shown. A slight increasing trend in both genders is observed.

**Fig 2:**
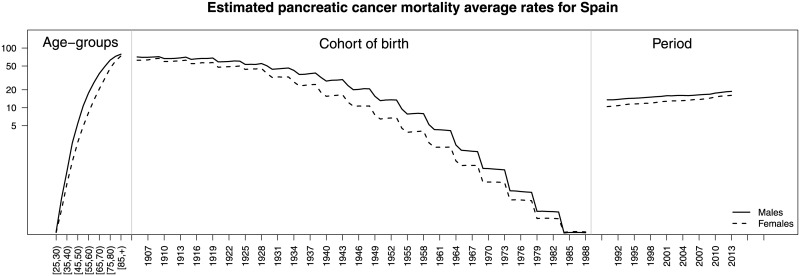
Estimated pancreatic cancer mortality average rates in whole Spain for males (continuous lines) and females (dotted lines) by age (left), birth cohort (center), and period (right). Posterior medians were used to compute rates. A semi-logarithmic scale was used on the y axis.

Fig 2 displays average estimated rates (${\hat{r}}_{gait}$) by age (left), cohort (center), and period (right) for whole Spain and both gender categories. This figure shows gender differences. The maximum differences between males and females in average rates by age groups is attained at the age groups [65, 70), [70, 75), and [75, 80) (left picture) where differences are about 16, 18, and 16 deaths per 100,000 respectively; differences in average rates by birth cohorts between males and females (central picture) are higher in those individuals born between the forties and the sixties approximately. In general, rates are lower for younger cohorts in both genders. Finally, although average rates increase with time, the difference between males and females seems to decrease (right picture).

**Fig 3:**
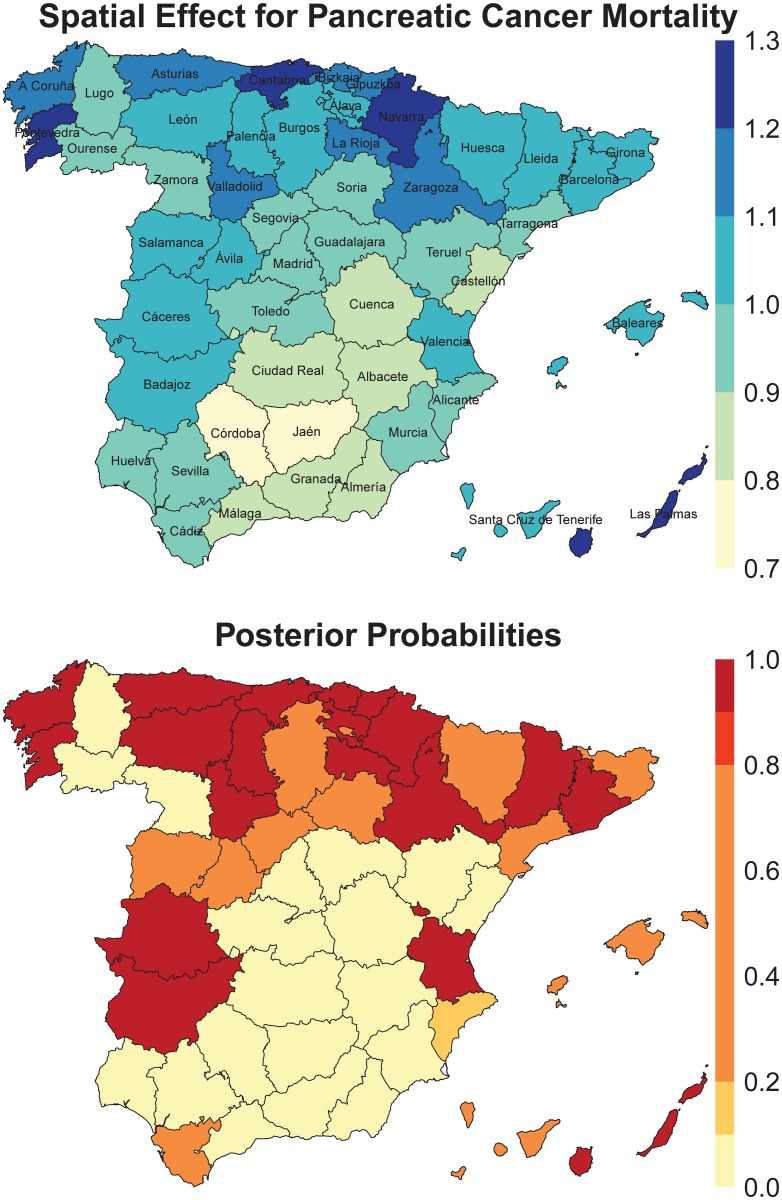
Region-specific effects (top), $e^{{\hat{\phi }}_{a}}$ (posterior medians), and posterior probabilities that the region effect is greater than one, $P(e^{{\hat{\phi }}_{a}}>1)$ (bottom).

In Fig 3, the region-specific effects (top), $e^{{\hat{\phi }}_{a}}$, together with the posterior probabilities that the region effects are greater than one $P(e^{{\hat{\phi }}_{a}}>1)$ (bottom) are displayed. The name of the provinces are included in the top map for an easier reading and interpretation of the results in this section. In general, the map shows a south-north geographical pattern, with higher significant effects in many regions located in the North of Spain (see, for example Navarra, Cantabria, Gipuzkoa, Bizkaia). Cáceres and Badajoz (in the south-west), Valencia, and Barcelona (in the Mediterranean area), and Las Palmas (a province in the Canary Islands) also contribute significantly to increase their final mortality rates.

**Fig 4:**
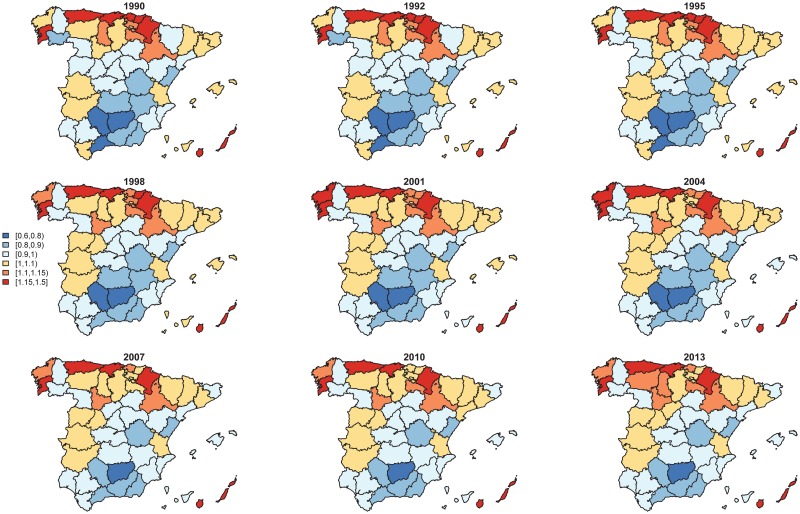
Spatial and spatio-temporal effects together, i.e., $e^{{\hat{\phi }}_{a}+{\hat{\delta }}_{at}}$.

Fig 4 shows the spatial and spatio-temporal effects together, i.e., $e^{{\hat{\phi }}_{a}+{\hat{\delta }}_{at}}$. Little changes of the geographical pattern have been observed over time. However, model selection criteria have pointed to retaining the interaction term in the final model.

**Fig 5:**
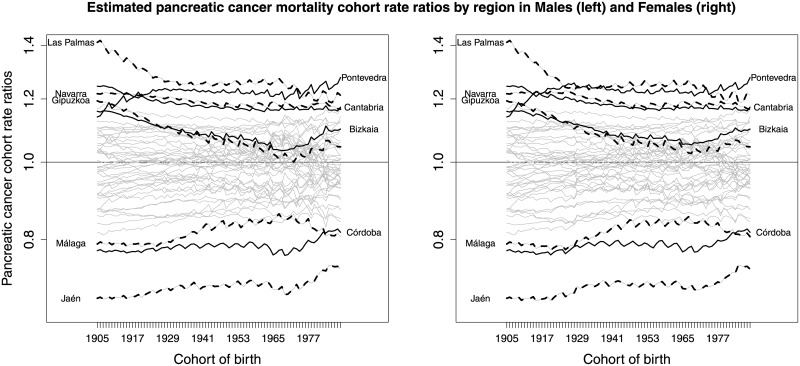
Ratios of region-specific average rates by birth cohort relative to the average rates by birth cohort for whole Spain (males on the left and females on the right).

In Fig 5, the ratios of region-specific average rates by birth cohort relative to the average rates by birth cohort for whole Spain are displayed for males (left) and females (right). These ratios are computed by dividing the region-specific average cohort rates by the estimated average cohort rate for whole Spain (see Fig 2, center). Looking at Fig 5, it is clear that Las Palmas, Cantabria, Navarra, Gipuzkoa and some other provinces in the North of Spain have higher average cohort rates than the average of Spain. On the other hand, provinces in the South (Jaen, Córdoba, Málaga, etc.) are the ones with lowest rates. Results are similar for both genders.

**Fig 6:**
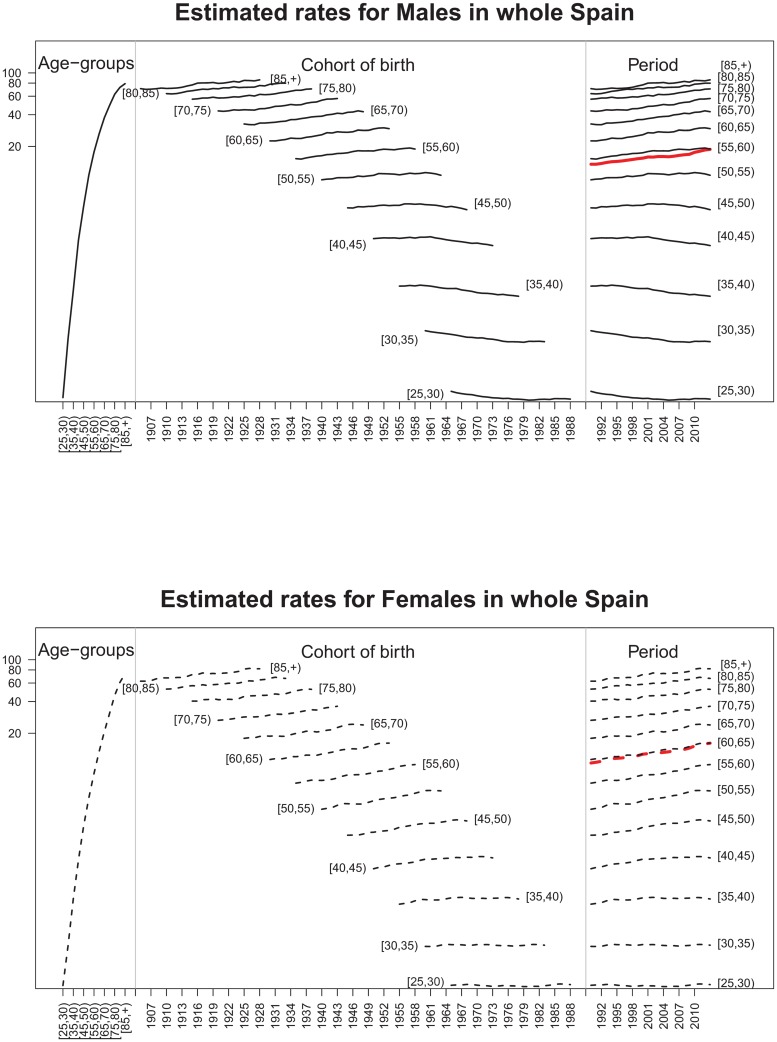
Age-specific pancreatic cancer mortality rates (on a semi-logarithmic scale on the y-axis) by birth cohort and period for whole Spain.

Finally, estimated rates by age-group, estimated age-specific pancreatic cancer mortality rates averaged by birth cohort, and estimated age-specific pancreatic cancer mortality rates averaged by period (in a semilogarithmic scale in the y-axis) are provided in Fig 6 for whole Spain. The left-hand side figure corresponds to males whereas the right-hand side plot refers to females. Each graph in the figure is divided into three panels. The one on the left displays the evolution of rates with age. The central panel shows the evolution of rates with the birth cohort by age groups, and the panel on the right displays the temporal evolution of rates by age groups. From this figure, it is clear that pancreatic cancer mortality rates increase with age (left panels). It is also clear that for males and females aged 60 years or older, the highest mortality rates correspond to the youngest cohorts of birth (central panels). That is, for the oldest age groups, rates increase with the cohort of birth. For example, the central panel of the left graph in Fig 6 represents the male rates for whole Spain by age group versus the cohort of birth. Here it is observed that the estimated rate for males aged between 70 and 75 years was slightly higher than 40 deaths per 100,000 in 1919. However, the rate for males aged between 70–75 years was around 60 deaths per 100,000 in 1943. In general, rates increase with the birth cohort for males and females aged 55 years or older. However, the behaviour of rates according to the birth cohort is different for males and females younger than 55 years. While a decrease is observed for males, a slight increase is observed for females. Something similar happens with the temporal evolution of rates (right panels in the graphs). In general, temporal rate trends increase for the oldest age groups in both genders. However, decreasing trends are observed for young males whereas stable or increasing trends are observed for young females. The average trend by age group displayed in red is also increasing for whole Spain and the three selected provinces. For interested readers, note that the same figures are also provided for three selected provinces in Fig A in S1 Appendix. The provinces were selected according to their geographical location. Navarre in the North (first row), Barcelona in the East (second row), and Las Palmas in Canary Islands (bottom row).

## Discussion

In this paper a multivariate spatial, gender, age, period, and birth cohort model is used to analyze pancreatic cancer rates in the provinces of Spain. The model allows to estimate rates taking into account spatial, temporal, age, and cohort effects as well as their possible interactions. Model fitting and inference has been carried out using the INLA methodology implemented in the R-INLA package, speeding up computations. To overcome model identifiability issues, several constraints have been considered during the estimation. Sensitivity analysis on the final selected model was also performed changing the prior distributions of the precision parameters. The final rates and the posterior distributions of all parameters (*β*_*g*_ and precisions) were robust to the choice of different hyper-prior distributions.

Our results indicate that average pancreatic cancer rates increase with age and time for both genders, whereas rates decrease with the birth cohort. The global temporal trends are increasing for both males and females. Although the rates are higher in men than in women, differences become smaller for the most recent years (see Fig 2). Our findings are consistent with the results given in [5]. These authors indicate that the pancreatic cancer mortality rates increase considerably in both genders since 1980. The average rates by age, cohort, and period are higher in males than females. Regarding age, the highest differences in rates between males and females are observed for the age group [65, 70), [70, 75), and [75, 80). The average cohort rates show an interesting pattern, the greatest difference between men and women being observed in those born between 1940–1960. From there on, the younger the birth cohort is, the smaller the difference becomes (see Fig 2). Some cohort differences are also identified by regions. Differences by gender are observed in the evolution of rates by age group if the time axis is the birth cohort (see central panel of Fig 6 for the whole Spain and central panels of Fig A in S1 Appendix—for specific provinces). For the youngest age groups, decreasing trends are observed for males whereas increasing trends are observed for females. The spatial pattern indicates a North-South pattern of pancreatic cancer mortality in Spain, being the provinces in the North the ones with higher effects on mortality during the studied period. Finally, the space-time evolution shows that the space pattern has changed little over time.

Due to its poor prognosis, pancreatic cancer is one of the malignant tumors that produces the highest rates of mortality worldwide [16]. According to data provided by different Spanish population-based registries in the latest available period (2003–2007), the highest adjusted incidence rates were observed in the provinces of La Rioja and Navarra in males, and in Navarra and Tarragona in females [17]. This is consistent with our mortality results. However, this geographical pattern is difficult to justify since it is very similar in men and women [18, 19] and it has been fairly stable over time. It seems to have its origin in unidentified environmental exposures. The largest exogenous risk factor identified, tobacco smoking, does not explain this spatial distribution. Unfortunately, the etiology of pancreatic cancer is generally unknown [20]. As in many other tumors, it is believed that its etiology is multifactorial, and a large number of risk factors have been identified in the literature.

Age-specific rates by year of birth for whole of Spain and provinces (Fig 6 and Fig A in S1 Appendix) show a characteristic evolution (javelin-like effect) also observed in colorectal [21] and stomach cancer [22]. The evolution of mortality may be due to the influence of smoking and diet.

Smoking would partially explain the mortality level off in the most recent generations, whereas the evolution of the prevalence of smokers would explain the differential risk between men and women [23]. With respect to diet, there is limited evidence that fruits provide protection against pancreatic cancer, and inconsistent evidence regarding vegetables. There is also suggestive evidence of increasing risk associated with red and processed meat, food and beverages containing fructose, and saturated fatty acids [24]. Risk of pancreatic cancer increases with heavy alcohol consumption and increasing body mass index [25]. Part of the differential risk in sex could be attributed to occupational exposure to various agents, including some pesticides, organic solvents, polycyclic aromatic hydrocarbons or nickel compounds described in the literature. Although all of the risk factors together could explain a large percentage of pancreatic cancer cases, individually most of these factors only explain a modest percentage of pancreatic cancer mortality cases [26]. Among the exogenous risk factors, it seems that tobacco consumption is the main one, and with lower degree of evidence, infection with Helicobacter pylori, type II diabetes, obesity, history of chronic pancreatitis, alcohol consumption, and occupational exposures already commented [17].

Genetic factor could contribute to a little percentage of the mortality cases, but interaction genes-environment become an important research field. Rare, moderately- to highly-penetrant mutations account for a small fraction of the familial aggregation of pancreatic cancer [24]. Based upon the hypothesis that common genetic variants contribute to susceptibility of common diseases such as cancer, there are numerous genome-wide association studies (GWAS). Those revealed several of the new loci harbor plausible candidate genes implicated in pancreas development, pancreatic beta-cell function, and predisposition to diabetes [19].

## Supporting information

S1 Appendix In this appendix, age-specific pancreatic cancer mortality rates (on a semi-logarithmic scale on the y-axis) by birth cohort and period for Navarra (first row), Barcelona (second row), and Las Palmas (bottom row) are given in Fig A. Besides, a detailed definition of Model (1), prior distributions, sensitivity analysis, and identifiability issues of APC models are given. The sensitivity analysis was performed to evaluate if the results were sensitive to the use of particular priors (in our case PC-priors). In particular, improper uniform priors on the standard deviations were also considered. Posterior means and standard deviations for the precision parameters were computed, and they are displayed in Table A. Fig B shows a dispersion plot of estimated rates with both sets of priors for the hyperparameters (first graph), and the posterior distribution of the precisions parameters with both set of priors showing that the results are essentially the same.
(PDF)

## References

[1] Ferlay J, Soerjomataram I, Dikshit R, Eser S, Mathers C, Rebelo M, et al Cancer incidence and mortality worldwide: Sources, methods and major patterns in GLOBOCAN 2012. Int J Cancer. 2015;136(5):E359–E386. 10.1002/ijc.29210 25220842

[2] American Cancer Society. www.cancer.org;. Available from: http://www.cancer.org/cancer/pancreaticcancer/index.

[3] Instituto de Salud Carlos III. Interactive Epidemiological Information System (ARIADNA);. Available from: http://ariadna.cne.isciii.es/MapP/.

[4] Hariharan D, Saied A, Kocher H. Analysis of mortality rates for pancreatic cancer across the world. Hpb. 2008;10(1):58–62. 10.1080/13651820701883148 18695761 PMC2504856

[5] Cabanes A, Vidal E, Aragones N, Pérez-Gómez B, Pollán M, Lope V, et al Cancer mortality trends in Spain: 1980–2007. Ann Oncol. 2010;21(Supplement 3):iii14–iii20. 10.1093/annonc/mdq089 20427355

[6] Rosso T, Malvezzi M, Bosetti C, Bertuccio P, Negri E, La Vecchia C. Cancer mortality in Europe, 1970–2009: an age, period, and cohort analysis. European Journal of Cancer Prevention. 2016;. 10.1097/CEJ.0000000000000282 27472086

[7] López-Abente G, Ramis R, Pollán M, et al Atlas municipal de mortalidad por cáncer en España, 1989–1998. Instituto de Salud Carlos III, Madrid; 2006.

[8] Eurostat. Revised European Standard Population 2013 (2013 ESP); 2012. Available from: http://www.ons.gov.uk/ons/guide-method/user-guidance/health-and-life-events/revised-european-standard-population-2013--2013-esp-/index.html.

[9] Papoila AL, Riebler A, Amaral-Turkman A, São-João R, Ribeiro C, Geraldes C, et al Stomach cancer incidence in Southern Portugal 1998–2006: a spatio-temporal analysis. Biom J. 2014;56(3):403–15. 10.1002/bimj.201200264 24596314

[10] Rue H, Martino S, Chopin N. Approximate Bayesian inference for latent Gaussian models by using integrated nested Laplace approximations. J R Stat Soc Ser B Stat Methodol. 2009;71(2):319–392. 10.1111/j.1467-9868.2008.00700.x

[11] Gelman A, Hwang J, Vehtari A. Understanding predictive information criteria for Bayesian models. Stat Comput. 2013;24(6):997–1016. 10.1007/s11222-013-9416-2

[12] Spiegelhalter D, Best N, Carlin B, Van Der Linde A. Bayesian measures of model complexity and fit. J R Stat Soc Ser B Stat Methodol. 2002;64(4):583–616. 10.1111/1467-9868.00353

[13] Heuer C. Modeling of time trends and interactions in vital rates using restricted regression splines. Biometrics. 1997;53(1):161–177. 10.2307/2533105 9147590

[14] Besag J. Spatial interaction and the statistical analysis of lattice systems. J Roy Statist Soc Ser B. 1974;36:192–236.

[15] Knorr-Held L. Bayesian modelling of inseparable space-time variation in disease risk. Stat Med. 2000;19(17–18):2555–2567. 10.1002/1097-0258(20000915/30)19:17/18<2555::AID-SIM587>3.0.CO;2-# 10960871

[16] Ferlay, J and Soerjomataram, I and Ervik, M and Dikshit, R and Eser, S and Mathers, C and et al GLOBOCAN 2012 v1 0. Cancer Incidence and Mortality Worldwide: IARC CancerBase No. 11; 2013. Available from: http://globocan.iarc.fr. 10.1002/ijc.29210 25220842

[17] López-Abente G, Núñez O, Pérez-Gómez B, Aragonés N, Pollán M. La situación del cáncer en España: Informe 2015. Madrid: Instituto de Salud Carlos III. Madrid; 2015.

[18] López-Abente G, Escolar A, Errezola M. Atlas del Cáncer en España. Madrid: Instituto de Salud Carlos III. Madrid; 1984.

[19] Wolpin BM, Rizzato C, Kraft P, Kooperberg C, Petersen G, Wang Z, et al Genome-wide association study identifies multiple susceptibility loci for pancreatic cancer. Nat Genet. 2014;46:994–1000. 10.1038/ng.3052 25086665 PMC4191666

[20] Hidalgo M. Pancreatic cancer. N Engl J Med. 2010;(362):1605–1617. 10.1056/NEJMra0901557 20427809

[21] López-Abente G, Ardanaz E, Torrella-Ramos A, Mateos A, Delgado-Sanz C, Chirlaque M. Changes in colorectal cancer incidence and mortality trends in Spain. Ann Oncol Off J Eur Soc Med Oncol ESMO. 2010;21(Suppl 3):iii76–82. 10.1093/annonc/mdq091 20427364

[22] Seoane-Mato D, Aragonés N, Ferreras E, García-Pérez J, Cervantes-Amat M, Fernández-Navarro P, et al Trends in oral cavity, pharyngeal, oesophageal and gastric cancer mortality rates in Spain, 1952–2006: an age-period-cohort analysis. BMC Cancer. 2014;14:254 10.1186/1471-2407-14-254 24725381 PMC4022416

[23] León-Gómez B, Colell E, Villalbí J, Barrio G, Domingo-Salvany A. Impact of smoke-free regulations on smoking prevalence trends in Spain. Eur J Public Health. 2016;. 10.1093/eurpub/ckw151 28177493

[24] World Cancer Research Fund / American Institute for Cancer Research. Food, Nutrition, Physical Activity, and the Prevention of Cancer: a Global Perspective. Washington DC: AICR; 2007.

[25] Klein A. Genetic susceptibility to pancreatic cancer. Mol Carcinog. 2012;51:14–24. 10.1002/mc.20855 22162228 PMC3570154

[26] Maisonneuve P, Lowenfels AB. Risk factors for pancreatic cancer: a summary review of metaanalytical studies. Int J Epidemiol. 2015;28(44):186–198. 10.1093/ije/dyu240 25502106

