# Supplementary material for: Spatial gender-age-period-cohort analysis of pancreatic cancer mortality in Spain (1990–2013)
Source: PLoS One. 2017 Feb 15;12(2):e0169751. doi: 10.1371/journal.pone.0169751 (PMC5310874; doi:10.1371/journal.pone.0169751)
Supplement: S1 Appendix — Besides, a detailed definition of Model (1), prior distributions, sensitivity analysis, and identifiability issues of APC models are given. The sensitivity analysis was performed to evaluate if the results were sensitive to the use of particular priors (in our case PC-priors). In particular, improper uniform priors on the standard deviations were also considered. Posterior means and standard deviations for the precision parameters were computed, and they are displayed in Table A. Fig B shows a dispersion plot of estimated rates with both sets of priors for the hyperparameters (first graph), and the posterior distribution of the precisions parameters with both set of priors showing that the results are essentially the same. (PDF) [file pone.0169751.s001.pdf]

S1 Appendix for "Spatial gender-age-period-cohort analysis of pancreatic cancer mortality in Spain (1990-2013)"

In this appendix, age-specific pancreatic cancer mortality rates (on a semi-logarithmic scale on the y-axis) by birth cohort and period for Navarra (first row), Barcelona (second row), and Las Palmas (bottom row) are given in Fig A. Besides, a detailed definition of Model 1, prior distributions, sensitivity analysis, and identifiability issues of APC models are given.

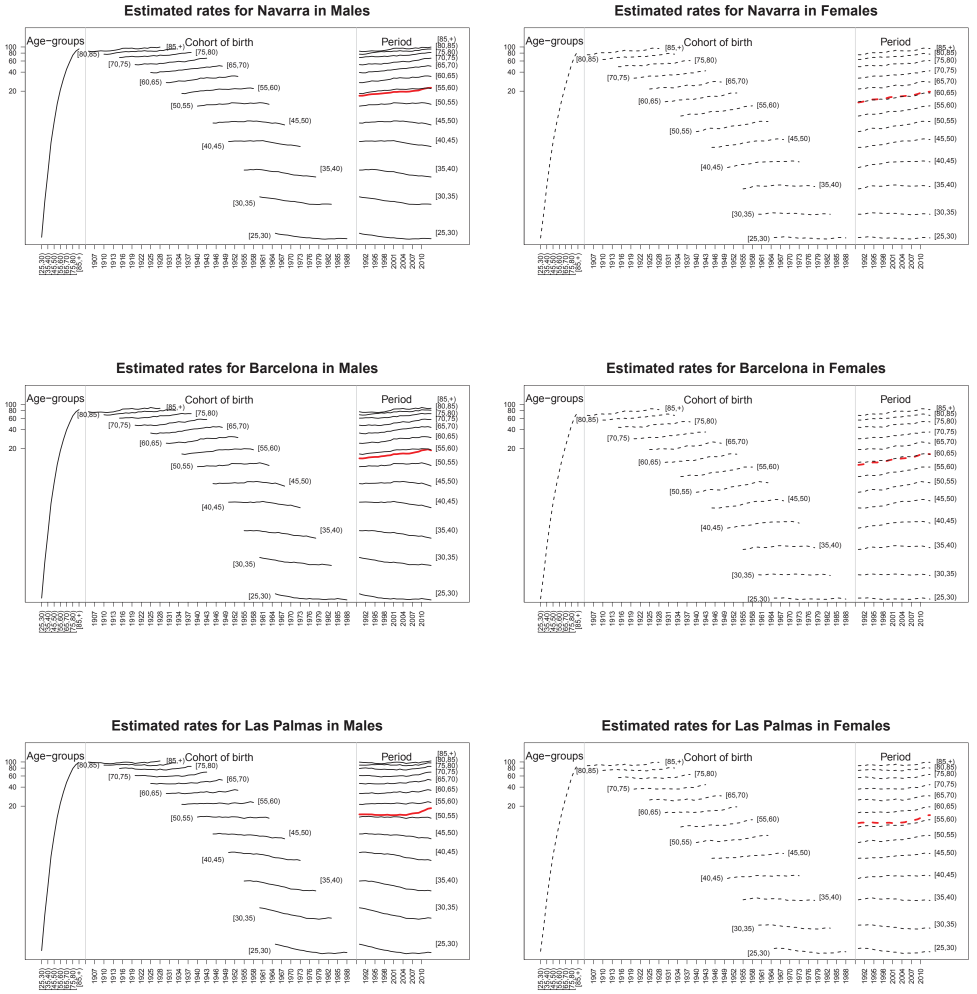

Fig A. Age-specific pancreatic cancer mortality rates (on a semi-logarithmic scale on the y-axis) by birth cohort and period for Navarra (first row), Barcelona (second row), and Las Palmas (bootom row).

Model 1 can be expressed in matrix form as

$$\begin{aligned}\log(\mathbf{r}) &= (\mathbf{I}_2 \otimes \mathbf{1}_A \otimes \mathbf{1}_I \otimes \mathbf{1}_T)\boldsymbol{\beta} + (\mathbf{I}_2 \otimes \mathbf{1}_A \otimes \mathbf{1}_I \otimes I_T)\boldsymbol{\alpha} + (\mathbf{I}_2 \otimes \mathbf{1}_A \otimes \mathbf{I}_I \otimes \mathbf{1}_T)\boldsymbol{\gamma} + \\ & (\mathbf{I}_2 \otimes \mathbf{1}_A \otimes \mathbf{K})\boldsymbol{\kappa} + (\mathbf{1}_2 \otimes I_A \otimes \mathbf{1}_I \otimes \mathbf{1}_T)\boldsymbol{\phi} + (\mathbf{1}_2 \otimes I_A \otimes \mathbf{1}_I \otimes I_T)\boldsymbol{\delta}.\end{aligned} \quad (1)$$

where $\boldsymbol{\beta} = (\beta_1, \beta_2)'$, $\boldsymbol{\alpha} = (\alpha_{11}, ..., \alpha_{1T}, \alpha_{21}, ..., \alpha_{2T})'$, $\boldsymbol{\gamma} = (\gamma_{11}, ..., \gamma_{1I}, \gamma_{21}, ..., \gamma_{2I})'$, $\boldsymbol{\kappa} = (\kappa_{11}, ..., \kappa_{1K}, \kappa_{21}, ..., \kappa_{2K})'$, $\boldsymbol{\phi} = (\phi_1, ..., \phi_A)'$, and $\boldsymbol{\delta} = (\delta_{11}, \delta_{12}, ..., \delta_{1T}, ..., \delta_{A1}, ..., \delta_{AT})'$. In this expression, $\otimes$ is the Kronecker product and $\mathbf{I}_2$, $\mathbf{I}_I$, $\mathbf{I}_A$ and, $\mathbf{I}_T$, represent identity matrices of dimension 2×2, $I \times I$, $A \times A$ and, $T \times T$ respectively. $\mathbf{1}_2$, $\mathbf{1}_I$, $\mathbf{1}_A$ and $\mathbf{1}_T$ represent vectors of ones of length 2, $I = 13$, $A = 50$, and $T = 24$ respectively, and finally, $\mathbf{K}$ is a matrix of dimension $(T \times I) \times K$ where $K = 84$ (the total number of cohorts is computed as $K = 5 \times (I - 1) + T$). This matrix is defined as follows: let us consider the first observation of our data set. This corresponds to the number of deaths for males in region 1, age-group 1, and year 1. For this observation, the cohort index would be $5 \times (13 - 1) + 1 = 61$ (it doesn't depend on gender or region index). Then, the first row of matrix K is a row of zeros except the value of column 61 which is 1. Suppose now that the second observation of our data set corresponds to the number of deaths in males of region 1, age-group 1, and year 2. For this observation the cohort index is $5 \times (13 - 1) + 2 = 62$, therefore the second row of matrix K is a row of zeros except the value of column 62 which is 1, and so on. In general, if the k-th row of the data set corresponds to the number of deaths and the population observed in age-group i and year t, then the k-th row of matrix K is a row of zeros except the value of the column $5 \times (13 - i) + t$ which is 1.

Finally, the random effects are assumed to follow the following multivariate normal distributions

$$\begin{aligned}\boldsymbol{\alpha} &\sim N(\mathbf{0}, \sigma^2_\alpha(\mathbf{I}_2 \otimes \mathbf{R}_\alpha)^-); \qquad \boldsymbol{\gamma} \sim N(\mathbf{0}, \sigma^2_\gamma(\mathbf{I}_2 \otimes \mathbf{R}_\gamma)^-); \\ \boldsymbol{\kappa} &\sim N(\mathbf{0}, \sigma^2_\kappa(\mathbf{I}_2 \otimes \mathbf{R}_\kappa)^-); \qquad \boldsymbol{\phi} \sim N(\mathbf{0}, \sigma^2_\phi \mathbf{R}_\phi^-); \\ \boldsymbol{\delta} &\sim N(\mathbf{0}, \sigma^2_\delta(\mathbf{R}_t \otimes \mathbf{R}_\phi)^-).\end{aligned}$$

In these expressions, $\mathbf{R}_\alpha$, $\mathbf{R}_\gamma$ and, $\mathbf{R}_\kappa$ are structure matrices corresponding to first order random walks for time, age, and cohort respectively (see for example [1], p. 95), and the symbol $^-$ denotes the Moore-Penrose generalized inverse. Note that a first order random walk is like a CAR model on a line where we use first order neighbors to define the structure matrix. Besides, $\mathbf{R}_\phi$ is the spatial neighborhood matrix defined by adjacency, where two areas are considered neighbors if they share a common border. Second order random walks (RW2) priors were also considered in the analysis but RW1 priors were finally selected by DIC and WAIC. Note also that a Type IV interaction [2] was chosen as the best option for the spatio-temporal interaction term, i.e., temporal trends are similar for neighboring regions. Finally, prior distributions on the precision parameters need to be given to fully specify the final selected model. Carroll and coauthors [3] stated that the default gamma priors on precision parameters in R-INLA could not be suitable in some cases in disease mapping models. Here, penalized complexity priors (PC-priors) [4] on the precision parameters ($\tau_\alpha = 1/\sigma^2_\alpha$, $\tau_\gamma = 1/\sigma^2_\gamma$, $\tau_\kappa = 1/\sigma^2_\kappa$, $\tau_\phi = 1/\sigma^2_\phi$ and $\tau_\delta = 1/\sigma^2_\delta$) were used. A vague zero mean normal prior with precision 0.001 was considered for the gender fixed effects.

A sensitivity analysis was performed to evaluate if the results were sensitive to the use of particular priors (in our case PC-priors). In particular, improper uniform priors on the standard deviations were also considered. Posterior means and standard deviations for the precision parameters were computed, and they are displayed in Table A. The results indicate that the posterior distributions of the precision parameters did not change much, and therefore PC-priors were chosen as they provided smaller values of WAIC and DIC.

Table A. Estimated posterior mean and standard deviation of the model parameters for different priors.

Parameter	PC-priors on precisions				Improper uniform priors on standard deviations			
	mean	sd	0.025quant	0.975quant	mean	sd	0.025quant	0.975quant
β_m	-9.448	0.0182	-9.485	-9.413	-9.449	0.0183	-9.486	-9.414
β_f	-9.915	0.0200	-9.956	-9.877	-9.916	0.0201	-9.957	-9.878
τ_t	1538.089	1380.232	361.7038	5160.176	1576.64	1468.8737	359.3415	5427.695
τ_i	1.429	0.385	0.7971	2.299	1.21	0.3646	0.6246	2.043
τ_k	129.293	50.195	56.2458	250.317	125.78	48.6994	54.9344	243.222
τ_a	66.684	14.918	41.6064	99.785	65.71	14.8276	40.8501	98.651
τ_{at}	1657.008	688.996	742.3417	3389.089	1640.19	680.7644	735.8873	3350.899

Finally, Fig B shows a dispersion plot of estimated rates with both sets of priors for the hyperparameters (first graph), and the posterior distribution of the precisions parameters with both set of priors. Results are essentially the same.
Identifiability problems are well-known in APC models [5, 6]. First of all, sum-to-zero constraints on the spatial, temporal, age, and birth cohort effects were applied to ensure the identifiability of the model intercept. To guarantee identifiability of the space-time interaction term and the main temporal and spatial effects the eigenvectors of the interaction precision matrix $\mathbf{R}_t \otimes \mathbf{R}_\phi$ (see Appendix 1) having null eigenvalues are used as linear constraints (see [7] for more details). However, the age, period, and cohort effects are still not identifiable (see for example [8–11]). Moreover, as these three effects (age, period, and cohort) vary across gender, then their relative effects of males versus females are not identifiable either [12]. However, as the linear predictor can always be identified and interpreted [13], the posterior means of pancreatic cancer mortality rates were computed and averaged by age, period, and cohort for each gender category.

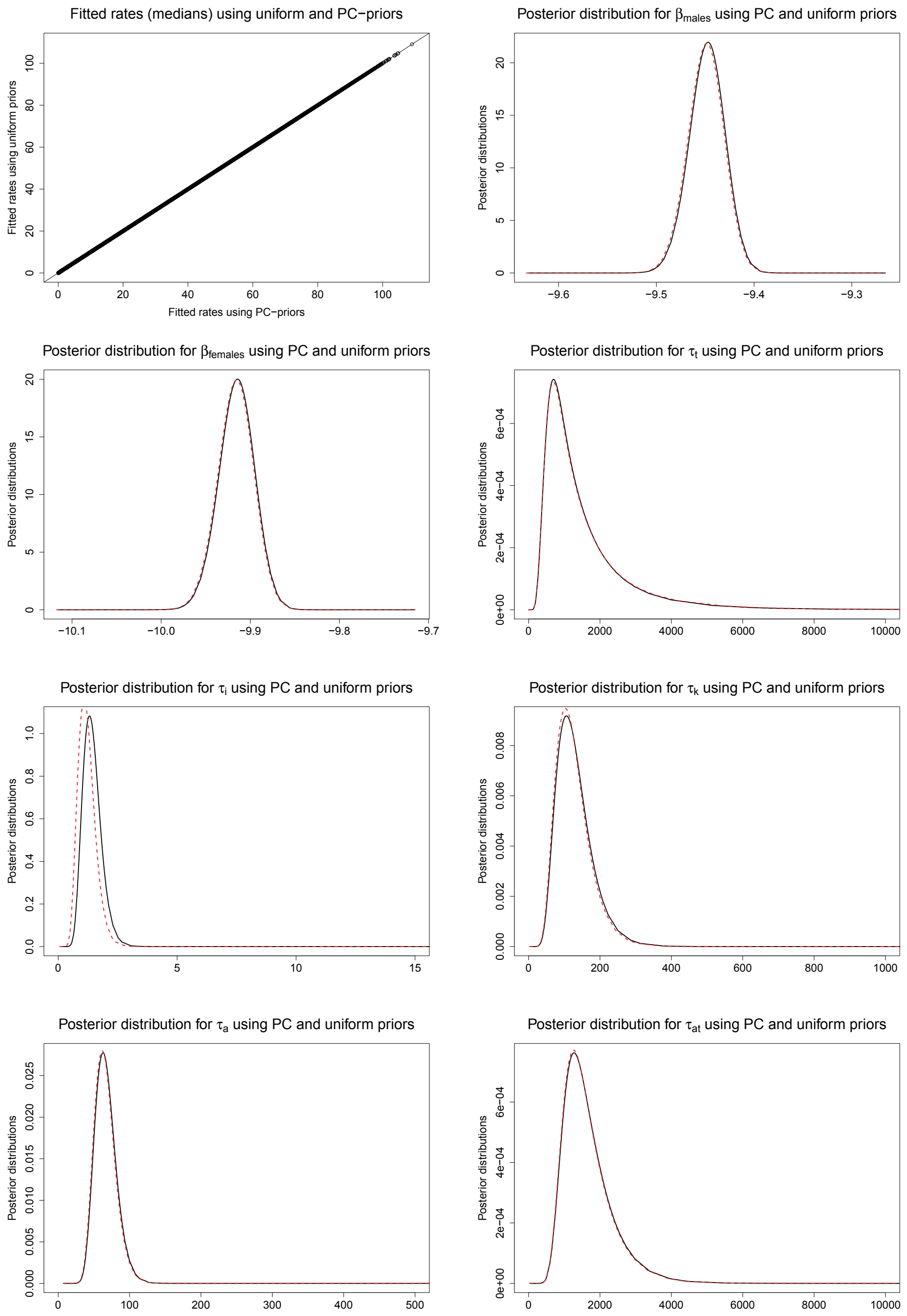

Fig B. Dispersion plot of estimated rates, and posterior distributions for fixed effects and hyperparameters using PC-priors on precisions (black lines) and improper uniform distributions on standard deviations (red lines).

References

1. Rue H, Held L. Gaussian Markov random fields: theory and applications. CRC Press; 2005.

2. Knorr-Held L. Bayesian modelling of inseparable space-time variation in disease risk. Stat Med. 2000;19(17-18):2555–2567.

3. Carroll R, Lawson AB, Faes C, Kirby R, Aregay M, K W. Comparing INLA and OpenBUGS for hierarchical Poisson modeling in disease mapping. Spat Spatiotemporal Epidemiol. 2015;14-15:45–54. doi:10.1016/j.sste.2015.08.001.

4. Simpson D, Martins T, Riebler A, et al. Penalising model component complexity: A principled, practical approach to constructing priors. arXiv Prepr arXiv14034630. 2014;.

5. Clayton D, Schifflers E. Models for temporal variation in cancer rates, II: Age-period-cohort models. Statistics in Medicine. 1987;6(4):469–481. doi:10.1002/sim.4780060405.

6. Held L, Riebler A. Comment on" Assessing Validity and Application Scope of the Intrinsic Estimator Approach to the Age-Period-Cohort (APC) Problem". Demography. 2013;50(6):1977.

7. Papoila AL, Riebler A, Amaral-Turkman A, São-João R, Ribeiro C, Geraldes C, et al. Stomach cancer incidence in Southern Portugal 1998-2006: a spatio-temporal analysis. Biom J. 2014;56(3):403–15. doi:10.1002/bimj.201200264.

8. Lagazio C, Biggeri A, Dreassi E. Age-period-cohort models and disease mapping. Environmetrics. 2003;14(5):475–490. doi:10.1002/env.600.

9. Schmid V, Held L. Bayesian extrapolation of space-time trends in cancer registry data. Biometrics. 2004;60(4):1034–1042. doi:10.1111/j.0006-341X.2004.00259.x.

10. Schmid V, Held L. Bayesian Age-Period-Cohort Modeling and Prediction - BAMP. Journal of Statistical Software. 2007;21(1):1–15. doi:10.18637/jss.v021.i08.

11. Riebler A, Held L, Rue H, Bopp M. Gender-specific differences and the impact of family integration on time trends in age-stratified Swiss suicide rates. Journal of the Royal Statistical Society: Series A (Statistics in Society). 2012;175(2):473–490. doi:10.1111/j.1467-985X.2011.01013.x.

12. Riebler A, Held L. The analysis of heterogeneous time trends in multivariate age-period-cohort models. Biostatistics. 2010;11(1):57–69. doi:10.1093/biostatistics/kxp037.

13. Holford TR. Age–Period–Cohort Analysis. In: Encyclopedia of Biostatistics. John Wiley and Sons, Ltd; 2005.Available from: `http://dx.doi.org/10.1002/0470011815.b2a03003`.
